# Impacted Incus Foreign Body in the Eustachian Tube

**Published:** 2019-03

**Authors:** Kartik Parelkar, Devika Shere, Ankur Wallic, Varun Dave, Karthik Rao

**Affiliations:** 1 *Department of * *Otorhinolaryngology* *, Grant Government Medical College and Sir J.J Hospitals, Mumbai, India.*; 2 *Department of * *Otorhinolaryngology* *, Durgabai Deshmukh Hospital, Hyderabad, India.*; 3 *Department of * *Otorhinolaryngology* *, Sion Hospital, Mumbai, India.*

**Keywords:** Eustachian tube, Foreign body, Middle ear, Endoscopy

## Abstract

**Introduction:**

Foreign bodies in the external auditory canal are fairly common, and tend to be seeds, toys, or insects. However, foreign bodies in the middle ear are rarer and are generally accidental or iatrogenic. Although there are numerous reports regarding fascinating foreign bodies in the ear, the same cannot be said about foreign bodies in the Eustachian tube (ET).

**Case Report:**

A 20-year-old male patient presented with right-sided active squamosal chronic otitis media (COM) persisting for 10 years. An incidental finding of a hyperintense foreign body (FB) located near the isthmus of the right ET was reported on high-resolution computed tomography (HRCT) of the temporal bones. The FB was bony (eroded incus), which was impacted near the isthmus of the ET and was extremely difficult to remove. A natural FB such as an eroded ossicle in the ET which aggravated the adhesive otitis and caused active squamosal COM has not been reported in the literature.

**Conclusion:**

We have reported this case not only for its rarity but also to make readers aware of such incidental findings which can be found only by imaging, and to suggest how they can be tackled with current equipment and technology.

## Introduction

An incidental finding of a hyperintense foreign body (FB), located near the isthmus of the right Eustachian tube (ET) was reported on imaging of the temporal bones in one of our patients with active chronic otitis media (COM). Minimal working space and compromised visual field make surgeries in the region of the ET challenging. Foreign bodies lodged in the ET are often missed, and are difficult to manage even if they are diagnosed.

## Case Report

**Fig 1:**
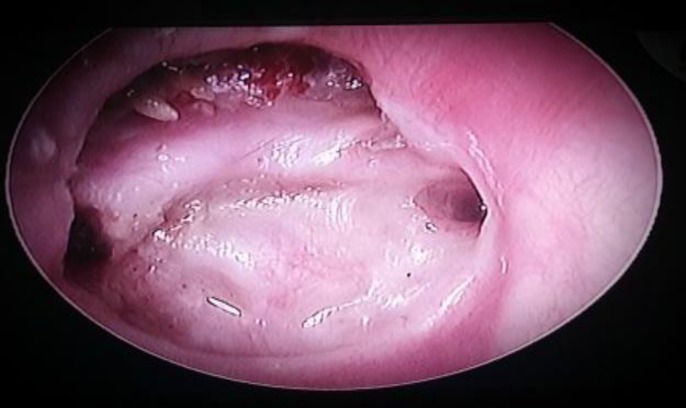
Right-ear otoendoscopy showing the small perforation near the Eustachian tube on the right side

A 20-year-old male patient presented to our out-patient department with right-sided ear discharge persisting for 10 years. The discharge was typically mucopurulent, scant to moderate, continuous, foul-smelling, occasionally blood-tinged and associated with hearing loss, suggestive of active squamosal COM. He gave no history of topical ear drops reaching the throat. On otoendoscopy there was erosion of the scutum, and the pars tensa was adherent over the promontory with no evidence of any ossicles. The tympanic segment of the fallopian canal was visible with the drum draped over it and a small perforation was noted in the antero-superior quadrant near the opening of the ET (Fig. 1). There were no clinical findings related to complications such as facial palsy, dizziness, tinnitus or cranial neuropathy. The opposite ear was normal.

**Fig 2:**
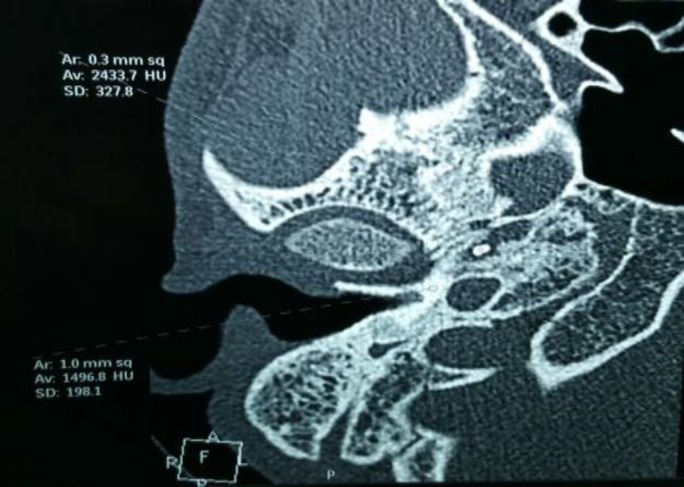
High-resolution computed tomography of the temporal bone showing the foreign body in the right Eustachian tube

The Rinne’s test was negative for all three frequencies (256,512,1,024 Hz) on the right side and negative for 256 Hz only on the left side. The Weber’s test with 512 Hz was lateralized to the left side. A pure tone audiogram revealed profound mixed hearing loss on the right side (100 dB HL) and mild conductive hearing loss on the left side (31 dB HL). High-resolution computed tomography (HRCT) temporal bone revealed thinning of the tegmen with the cholesteatoma sac occupying the mastoid cavity and no visible ossicles. It also reported an incidental FB (metallic or bony) with an intensity of 2,400 HU located at 7.2 mm from the ET opening in the middle ear and measuring 1.3 × 2.2 mm in size (Fig.2).

A screening MRI showed no artifact and favored the diagnosis of a sclerotic bony FB. After informed consent, diagnostic nasal endoscopy and routine investigations indicated that the patient was fit for surgery. He underwent surgery under local anesthesia with 2% lignocaine and adrenaline, supplemented by intravenous sedatives. 

A right-sided Wilde’s incision was taken, and temporalis fascia and tragal cartilage grafts were harvested. The FB in the ET was not visualized under a microscope. The tympanomeatal flap was elevated and a canal-wall-down mastoidectomy was performed. A small piece of Merocel^®^ soaked in 4% lignocaine and adrenaline was kept near the ET opening to prevent the bone dust from entering the ET.

**Fig 3:**
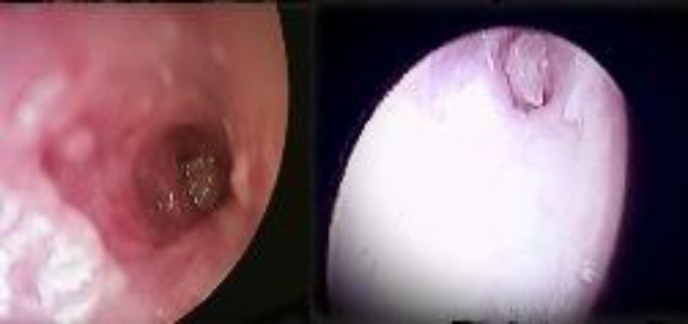
Right Eustachian tube view after canal-wall-down mastoidectomy with 4-mm zero-degree and 2.7-mm 30-degree scope showing the eroded incus in the Eustachian tube

After lowering the canal wall, the FB was visible with a 4-mm zero-degree endoscope but none of the routine otology instruments could manipulate the FB. A 2.7-mm pediatric 30-degree endoscope gave better working space, but compromised the visual field. However, using endoscopes we could visualize that the FB was an eroded ossicle (incus) (Fig. 3).

Multiple fine instruments were tried, which included a ball point, curved pick, and 2-mm flexible grasping forceps used in general surgery for gall bladder surgeries. The fine curved pick could reach the foreign body, but there was bleeding during manipulation. An infant feeding tube of the smallest size was used as suction. 

Unfortunately, the FB was displaced further medially into the ET. Endonasal clearance of secretions and blood from the ET was performed with a curved suction cannula. An attempt to suck out the FB from the nasopharyngeal end of the ET was made, but unfortunately the FB was not retrieved intra-operatively, and hence it was not possible to assess its histopathology.

We had anticipated this difficulty and designed a special graft which would keep the middle ear independent of the ET ventilation. 

**Fig 4:**
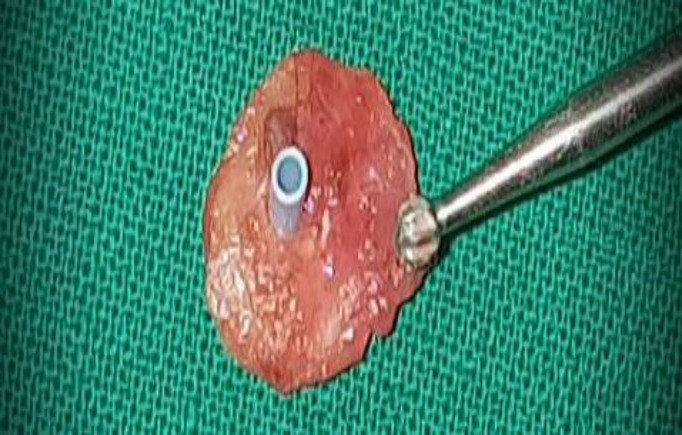
The cartilage T-tube graft

We used a Goode’s T-tube which was inserted through a small hole made in the tragal cartilage with a cutting burr. A small piece of temporalis fascia was then placed over the lateral side of the designed graft with the T-tube coming out through it (Fig. 4). 

The middle ear was filled with steroid-soaked gel foam, and the fashioned graft was adjusted over it in an underlay manner. The outer end of the small T-tube was trimmed to an appropriate size. The mastoid cavity and the ear canal was filled with Betadine®-soaked gel foam. 

**Fig 5:**
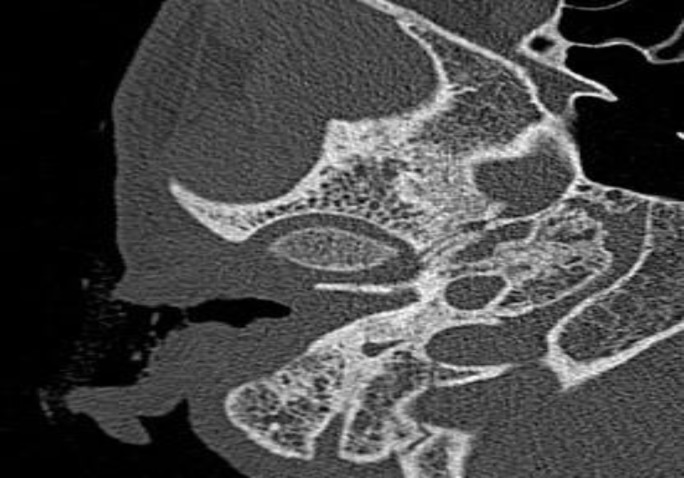
High-resolution computed tomography temporal bone post-operatively without the foreign body in the right Eustachian tube

A wide meatoplasty was performed and the wound was closed in layers. HRCT was repeated after 1 week and showed no evidence of FB in the ET. The patient remains asymptomatic, on a 6-month follow-up (Fig.5).

## Discussion

As described above, our patient was a typical case of active squamosal COM, and the FB was an incidental finding on imaging. If such findings are missed, these patients tend to have recurrence or failures after surgery. There have been a few previous reports of such instances. For example, Igarashi et al. published an interesting report regarding an acupressure metallic bead foreign body in the Eustachian tube. They too encountered difficulties during removal and displaced the bead deeper in the ET, and had to remove it after canal-wall-down procedure (1). Morita et al. also reported similar acupressure pellets going into the middle ear and ET, which they deemed too difficult to remove (2). Finally, Weber and Rosner reported a case with atelectatic ear drum with persistent ear discharge in which a displaced pressure equalization tube was found on HRCT in the right ET (3). Such objects generally become extruded into the external ear canal, but can occasionally cause such a complication too.

Imaging also helped us to understand the exact site and dimensions, and helped to plan our management strategy. It is extremely important to know about the erosion of the carotid artery canal especially when the surgery deals with the area near the ET. We suspect that recurrent COM led to erosion of incus, which over a period of time was displaced into the ET, aggravating the ET dysfunction. However, we did not see a way to confirm the sequence of these events. A similar report of a FB-induced cholesteatoma and external auditory canal granuloma has been described by Shui-Hong (4). They found a plastic stick intra-operatively, which was missed despite imaging the patient.

There have been reports of foreign bodies causing erosion of the carotid canal (5). Careful planning and correct execution is essential. We recommend the use of an endoscope for such cases owing to its superior field of vision and magnification. Although it might be easier to remove the FB located toward the tympanic orifice, there is a limitation in the choice of instruments dedicated to the procedures for operating in the ET. Hence, there are many cases in which the FB tends to become displaced deeper (1), including our case. Most cases with medially displaced FB in the ET require a canal-wall-down procedure to increase the working space and vision. An infant feeding tube of the smallest size for suction can be effectively used for atraumatic suctioning in the deeper part of the ET. Minimal manipulation is advisable to prevent edema and bleeding from obscuring the surgical field.

Despite all the preparation, it is good to have a back-up plan. We therefore had planned to fashion a graft which would ventilate the middle ear in case we were not able to remove the FB or if there was fibrosis in the ET due to the manipulation. Other reports in the literature have not mentioned any surgical options, in case one fails to remove the FB. Although it has been mentioned that drilling away the antero-inferior annulus improves the access (5), this is a risky option. An innovative minimally invasive approach to the ET for removal of deeper FB could be by using a sialo-endoscope, but there is no literature on this, and the view that a 4-mm zero-degree scope offers would have to be sacrificed. Finer and more flexible instruments are needed to carry out procedures in the ET.

## Conclusion

FB can be one of the causes of ET dysfunction and must not be ignored. Particularly in complicated ears, revision surgeries, and failure cases imaging may help to reveal a missed etiology. Endoscopic, minimally invasive, and atraumatic techniques are recommended. In case of failure to remove the FB, an aero-tympanoplasty, for prolonged middle-ear ventilation, independent of the ET can be performed.
